# Perceptions of instructor quality, loyalty and recommendation intentions in fitness centers: a comparative analysis by role and users characteristics

**DOI:** 10.3389/fspor.2026.1731975

**Published:** 2026-03-03

**Authors:** Francisco Campos, Daniela Almeida, José Aleixo, Diogo Aveiro, Viorel Petru Ardelean, Vlad Adrian Geantă, Fernando Martins

**Affiliations:** 1 Polytechnic University of Coimbra, Coimbra, Portugal; 2 SPRINT – Sport Physical activity and health Research & INnovation cenTer, Polytechnic University of Coimbra, Coimbra, Portugal; 3 Instituto de Telecomunicações – Delegação da Covilhã, Covilhã, Portugal; 4 LFitness, Oliveira do Bairro, Portugal; 5 Department of Physical Education and Sport Sciences, University of Limerick, Limerick, Ireland; 6 Department of Physical Education and Sport, Faculty of Physical Education and Sport, Aurel Vlaicu University of Arad, Arad, Romania; 7 inED – Center for Research and Innovation in Education, Polytechnic University of Coimbra, Coimbra, Portugal

**Keywords:** fitness centers, fitness service, loyalty, perceived quality, recommendation intention

## Abstract

**Background:**

Service quality is a critical determinant of satisfaction and behavioral intentions in fitness centers. Instructors play a pivotal role in shaping users’ perceived quality and, consequently, their loyalty and recommendation intentions. This study aimed to compare users’ ratings of quality of intervention, loyalty intention, and recommendation intention with instructors’ estimations of how users would rate these same dimensions, and to examine how users’ ratings vary according to sex, age, educational level, and gym attended.

**Methods:**

A total of 589 users and 145 instructors from five gyms within a Portuguese fitness chain completed an online questionnaire in which users rated quality of intervention, loyalty intention, and recommendation intention, while instructors reported their estimations of how users would rate these same dimensions, rather than providing their own evaluations. Independent samples *t*-tests and one-way ANOVA were performed to compare perceptions between users and instructors, and across user groups by sex, age, education level, and gym attended.

**Results:**

Users consistently rated instructor quality, loyalty, and recommendation intentions higher than instructors did. Among users, significant differences were found across age, education level, and gym attended: older and less-educated participants, and those from specific gyms, reported higher scores. Only recommendation intention differed by sex, with female users more frequently reporting that they would recommend the gym.

**Conclusion:**

This study highlights the crucial role of instructor quality in shaping satisfaction and behavioral intentions within fitness settings. The results emphasize the importance of reflective professional development for instructors and segmentation-based loyalty strategies for users. These findings offer actionable insights for fitness managers to tailor service delivery, strengthen customer loyalty, and foster sustainable organizational success in a competitive market.

## 1 Introduction

The fitness industry has evolved into a highly competitive and service-oriented sector where customer experience, rather than infrastructure alone, determines long-term success. Within this context, perceived service quality plays a decisive role in shaping customer satisfaction, loyalty, and retention (1, 2). In particular, the instructor's technical, relational, and communicative behaviors represent critical components of service delivery and strongly influence how users evaluate their overall fitness experience (3–7). Instructor behaviors such as pedagogical feedback and the ability to adjust instructional strategies to participants' needs have also been identified as determinants of perceived service quality and engagement (8, 9). As fitness trends continue to evolve globally (10, 11), understanding how such human-centered factors contribute to perceived quality has become a central concern for sport and fitness management.

A consistent body of evidence indicates that perceived service quality positively affects customer satisfaction and behavioral outcomes such as loyalty and recommendation intentions (12–15). In addition, perceived value has been highlighted as a complementary construct in this chain, often working alongside service quality and satisfaction to explain loyalty and recommendation intentions (14, 16). Specifically, quality attributes related to environment, reliability, or staff competence have been linked to favorable consumption behaviors (17, 18). Recent reviews reaffirm that higher service quality enhances satisfaction, which, in turn, promotes loyalty and customer retention, reinforcing organizational sustainability (19, 20). Furthermore, the intention to recommend a gym or instructor, often manifested through word-of-mouth communication, has been recognized as a key behavioral indicator of perceived value and satisfaction (16).

Instructors' interpersonal and technical skills are particularly relevant to this dynamic. Glaveli et al. (21) demonstrated that communication and empathy strengthen trust and satisfaction, while Barbosa et al. (22) found that both relational and technical-pedagogical competencies contribute significantly to perceived service quality. In addition, recent cross-cultural evidence suggests that the public image and behavioral expectations of fitness instructors may vary across countries and training systems (23), reinforcing the importance of contextualized professional standards and cultural awareness in service delivery. These findings suggest that instructor behavior, beyond exercise programming or technique, may play a decisive role in customer experience and retention. Yet, although prior studies have examined either user perceptions or instructor performance, comparative analyses involving both perspectives remain limited in the literature to date.

In addition, individual characteristics such as sex, age, and educational level appear to influence users' evaluations of instructor quality and service experience. Older users tend to report higher satisfaction and stronger loyalty, often valuing relational stability, trust, and interpersonal competence more than younger users, who are typically more demanding and less loyal (1, 24, 25). Gender differences have also been reported, with female users generally assigning greater importance to relational and communicative aspects of instructor behavior (1, 24, 26, 39). Likewise, educational level appears to influence service expectations, with more highly educated users displaying higher standards regarding technical competence, innovation, and communication quality (27). However, the extent to which these variables affect loyalty or recommendation intentions remains insufficiently understood. Addressing these gaps is particularly relevant for fitness managers and policy makers seeking to optimize human resource development, tailor interventions to client profiles, and improve service quality strategies in a sustainable manner.

Beyond examining users' perceptions alone, this study introduces a comparative approach that contrasts users' actual evaluations with instructors' meta-perceptions of how users experience service quality, loyalty, and recommendation intentions. This distinction is theoretically relevant, as instructors’ estimations represent second-order perceptions rather than self-evaluations, a perspective that remains underexplored in fitness service quality research. By identifying perceptual gaps between users and instructors, this study contributes to service quality and relationship management theory by highlighting potential misalignments that may influence professional self-regulation, reflective practice, and service improvement strategies in fitness centers.

Therefore, the present study aims (a) to compare fitness users' ratings of quality of intervention, loyalty intention, and recommendation intention with instructors’ estimations of how users evaluate these same dimensions; and (b) to examine how users' ratings of these constructs vary according to sex, age group, educational level, and gym attended. By integrating users' direct evaluations with instructors' perceptions of user experience, this study seeks to identify perceptual gaps and sociodemographic patterns that are relevant for service quality management in fitness centers. Understanding these perceptual differences is crucial for fitness managers to enhance service quality, strengthen customer loyalty, and promote long-term sustainability in the fitness industry.

Importantly, instructors' responses in the present study do not represent self-evaluations of their own performance, but rather estimations of how users perceive service quality, loyalty intention, and recommendation intention. From a theoretical perspective, these estimations can be understood as second-order perceptions or meta-perceptions, reflecting instructors' beliefs about users' evaluations. Examining such perceptual gaps between users and service providers has been highlighted as particularly relevant in service quality and relationship management research, as misalignments may influence professional self-regulation, communication strategies, and service delivery decisions in applied fitness contexts.

## 2 Materials and methods

### 2.1 Participants

All instructors and active users registered in any of the five gyms of the chain during the data collection period were invited to participate. The gyms belonged to the same fitness chain and were distributed across different cities within the same region of Portugal, operating under a common organizational and managerial framework. For instructors, the response rate was 90.60%. For users, due to continuous changes in membership status (e.g., new registrations, cancellations, and temporary suspensions), a precise response rate could not be calculated. Inclusion criteria for instructors were: (a) being at least 18 years old and (b) currently providing services in one of the five gyms. For users, the inclusion criteria were: (a) being 18 years of age or older and (b) being an active member of one of the five gyms during data collection period. No minimum length of membership for users or professional experience for instructors was required. Respondents who did not complete the questionnaire or did not provide informed consent were excluded from the analysis. All participants were informed about the purpose and objectives and provided informed consent. The study was conducted in accordance with the Declaration of Helsinki and was approved by the Ethics Committee of the Polytechnic University of Coimbra (approval number D40/2024).

### 2.2 Instruments

For data collection, a reduced and adapted version of the Qualidade do Instrutor de Fitness—Atividades de Grupo [Quality of the Fitness Instructor—Group Activities (QIF-AG)] questionnaire, of Campos et al. (28), was used. This version was constructed for the present study by selecting and adapting items from the original validated instrument, in order to fit the objectives and contexts of this research. The procedures reported below refer to the adaptation and application of the instrument in the present study. This was produced by the research group, in collaboration with the fitness chain, so that the information collected corresponded to what was intended by all parties. Thus, four questionnaires were constructed, for group activities, personal training, exercise room and physical assessment services, each one with two versions: perception (vP), applied to the users; self-perception (vAP), to instructors.

After being constructed for online data collection, the questionnaires were submitted to a multistage validation process. First, the items were adapted from previously validated instruments used to assess perceived service quality and behavioral intentions in fitness and sport services (12, 14, 28–30). Second, content validity was assessed by two panels of independent experts with PhD degrees in Sport Sciences and extensive experience in service quality research and professional practice. The first panel feedback led to minor wording refinements while preserving the theoretical meaning of each construct; the second, agree with proposed changes, and considered the instruments valid for the objectives.

Although a specific first part, directed to different areas of intervention within fitness services, there are then some cross-sectional questions in the four questionnaires, with slight differences in their formulation depending on the version associated with the respondent [vP (users) or vAP (instructors)]. The constructs analyzed were quality of intervention, loyalty intention, and recommendation intention. All items were answered on a 7-point Likert scale, where higher values indicate more positive evaluations. Quality of intervention was assessed with the item “In general, how do you evaluate the quality of the service provided by the instructors?”, ranging from 1 (extremely negative) to 7 (extremely positive). Loyalty intention was assessed with the item “How do you classify your loyalty intention at this moment?”, ranging from 1 (leave as soon as possible) to 7 (only for reasons of force majeure). At least, recommendation intention was assessed with the item “How much would you recommend this gym to a person you trust (e.g., family member, friend, spouse)?”, ranging from 1 (certainly not) to 7 (certainly yes). In the instructor version, the items were reformulated to ask them how they believe users would evaluate these dimensions; this item referred to instructors' estimations of how users evaluate the gym, rather than to the instructors' own opinions.

Although quality of intervention, loyalty intention, and recommendation intention were operationalized as single-item global indicators, previous research supports the adequacy of single-item measures for capturing concrete and unidimensional constructs, particularly in applied service contexts where respondent burden must be minimized. Single-item measures have demonstrated acceptable validity when the construct is concrete and clearly defined, as is the case with overall service quality, loyalty intention, and recommendation intention in fitness services.

### 2.3 Procedures

For the defined objectives, in a previous phase, the collection process was articulated with the coordination of the gym chain, which was in charge of framing the work and requesting the collaboration of all the instructors who provide services in the five gyms of the group. They assumed an identical role with the users; Although the data collection was done online, there was an awareness among users of the importance of their contribution, to improve the quality of the service provided.

For the distribution of the questionnaires to users and instructors, Salesforce's Customer Relationship Management (CRM) management software was used, which allowed that the specific instruments to be addressed according to the type of respondent (users or instructors). The data was collected for about a month, from November 25 to December 20, 2024. After being extracted to Microsoft Excel, were transferred to the Statistical Package for the Social Sciences (SPSS) (version 28) for the respective statistical analysis.

### 2.4 Statistical analysis

Given the real-world sampling context, group sizes were unequal across profiles and sociodemographic categories. However, the statistical procedures applied are robust to differences in group size, and all analyses met the required assumptions for group comparisons. Although different areas of activity were descriptively characterized, they were not used as segmentation criteria in the inferential analyses. The sampling strategy intentionally aimed to reflect the heterogeneous and natural composition of fitness services. Therefore, areas of activity were included to ensure ecological representativeness rather than analytical stratification.

Descriptive statistics were used for characterization, specifically *M* ± *SD* values. The independent Student *t*-test was used to compare the quality of intervention (QI), loyalty intention (LI), and recommendation intention (RI) between instructors and users, after checking the normality assumption (31, 32). The similar procedure was used to compare QI, LI and RI between sex of users. The one-way ANOVA was used to compare QI, LI and RI between users group age, education level and gym attended, after checking normality and homogeneity assumptions (33, 34). Tukey's *post-hoc* test was performed for multiple comparisons of means within groups (34).

For Student *t*-test, the classification of the effect size (ES) is performed through the reference values of Cohen's *d*, according to O'Donoghue (35): small (*d* < .20); moderate (.20 *≤* *d* < .80); large (*d* ≥ .80). In turn, for one-way ANOVA, the classification of the ES is made according to the reference values of eta squared (*η*^2^) (32): small (*η*^2^ ≤ .05); medium (.05 < *η*^2^ ≤ .25); high (.25 < *η*^2^ ≤ .50); very high (*η*^2^ > .50). Statistical analysis was performed using SPSS (version 28), for a significance level of 5% (*p* < .05).

## 3 Results

### 3.1 Participant characteristics

A total of 589 users and 145 instructors from five gyms belonging to a fitness chain in Portugal participated in the study. The sociodemographic and professional characteristics of the participants, including age, sex distribution, and areas of activity, are presented in Table 1.

**Table 1:** Sociodemographic and professional characteristics of users and instructors.

Characteristics	U (*n* = 589)	I (*n* = 145)
Age (M ± SD)	42.28 ± 12.98	26.79 ± 6.65
Sex (*n*; %)	F: 451; 76.57%	F: 51; 35.17%
	M: 138; 23.43%	M: 94; 64.83%
Areas of activity (*n*; %)	GA: 270; 45.84%	GA: 32; 22.07%
	PT: 60; 10.19%	PT: 40; 27.58%
	ER: 111; 18.85%	ER: 41; 28.28%
	PA: 148; 25.12%	PA: 32; 22.07%

U, users; I, Instructors; F, female, M, male; GA, group activities, PT, personal training, ER, exercise room, PA, physical assessment.

### 3.2 Differences between users and instructors ratings

Users' ratings of quality of intervention (QI), loyalty intention (LI), and recommendation intention (RI) were compared with instructors' estimations of how users would evaluate these same dimensions. Descriptive statistics and results from independent-samples *t-*tests are presented in Table 2.

**Table 2:** Characterization and comparison, between U and I perceptions, about the QI, LI and RI.

Dimensions	U (*n* = 589)	I (*n* = 145)	*p*	*d*
QI	6.11 ± 1.18	5.58 ± .82	.001*	.470
LI	5.95 ± 1.42	5.31 ± 1.01	.001*	.471
RI	6.03 ± 1.38	5.61 ± .93	.001*	.322

U, users; I, instructors; QI, quality of intervention, LI, loyalty intention, RI, recommendation intention; Variables measured on a 7-point Likert scale (1 = lowest; 7 = highest); *d* = Cohen's *d* (effect size).

* Significant for *p* < .05.

Results indicate significant differences between instructors and users regarding QI (*t* = 6.303; *p* = .001; *d* = .470, moderate ES), LI (*t* = 6.187; *p* = .001; *d* = .471, moderate ES) and RI (*t* = 4.373; *p* = .001; *d* = .322, moderate ES). In the three dimensions, the answer values of the users are higher than those of the instructors [QI: U (6.11 ± 1.18), I (5.58 ± .82); LI: U (5.95 ± 1.42), I (5.31 ± 1.01); RI: U (6.03 ± 1.38), I (5.61 ± .93)]. Although statistically significant, the effect sizes indicate a moderate difference between users' and instructors' ratings. Even small-to-moderate effects may be meaningful in applied fitness settings, where cumulative user experiences influence retention.

### 3.3 Differences in users ratings by sex, age group, education level, and gym attended

Differences in users' ratings of quality of intervention (QI), loyalty intention (LI), and recommendation intention (RI) were examined according to sex, age group, educational level, and gym attended. The results of these analyses are presented in Tables 3–6. Overall, users reported high levels of quality of intervention, loyalty intention, and recommendation intention, with mean values above the midpoint of the scale across all sociodemographic groups.

**Table 3:** Characterization and comparison according to sex, in users, of QI, LI and RI.

Dimensions	F (*n* = 451)	M (*n* = 138)	*p*	*d*
QI	6.13 ± 1.18	6.04 ± 1.18	.417	.079
LI	5.98 ± 1.36	5.84 ± 1.60	.316	.098
RI	6.10 ± 1.33	5.81 ± 1.52	.035*	.205

F, female; M, male; QI, quality of intervention; LI, loyalty intention; RI, recommendation intention; Variables measured on a 7-point Likert scale (1 = lowest; 7 = highest); *d* = Cohen's *d* (effect size).

* Significant for *p* < .05.

**Table 4:** Characterization and comparison according to group age, in users, of QI, LI and RI.

Dimensions	A1 (*n* = 22)	A2 (*n* = 108)	A3 (*n* = 124)	A4 (*n* = 179)	A5 (*n* = 98)	A6 (*n* = 58)	*p*	*η* ^2^
QI	6.40 ± .90	5.87 ± 1.23^a^	5.99 ± 1.34^b^	6.06 ± 1.22	6.31 ± 1.01	6.53 ± .79^a^^,^^b^	.003*	.030
LI	6.13 ± 1.24	5.79 ± 1.41^a^	5.80 ± 1.50^b^	5.94 ± 1.50	5.95 ± 1.40	6.51 ± .90^a^^,^^b^	.033*	.021
RI	6.09 ± 1.68	5.85 ± 1.31^a^	5.87 ± 1.57^b^	6.03 ± 1.40	6.12 ± 1.31	6.56 ± .72^a^^,^^b^	.026*	.022

*η*^2^ = eta squared (effect size); A1, Less than 21 years old; A2, 21–30; A3, 31–40; A4, 41–50; A5, 51–60; A6, More than 60; QI, quality of intervention; LI, loyalty intention; RI, recommendation intention; Variables measured on a 7-point Likert scale (1 = lowest; 7 = highest); multiple comparison using *post-hoc* test: ^a^A2 vs. A6, ^b^A3 vs. A6.

* Significant for *p* < .05.

**Table 5:** Characterization and comparison according to education level, in users, of QI, LI and RI.

Dimensions	E1 (*n* = 55)	E2 (*n* = 166)	E3 (*n* = 254)	E4 (*n* = 114)	*p*	*η* ^2^
QI	6.49 ± .97^a^	6.22 ± 1.04	5.96 ± 1.27^a^	6.09 ± 1.21	.012*	.019
LI	6.25 ± 1.41^b^	6.17 ± 1.26^c^	5.85 ± 1.44	5.70 ± 1.52^b^^,^^c^	.011*	.019
RI	6.30 ± 1.33	6.13 ± 1.24	5.99 ± 1.40	5.85 ± 1.52	.158	.009

*η*^2^ = eta squared (effect size); E1, 9th grade; E2, 12th grade; E3, BSc; E4, MSc or PhD; QI, quality of intervention; LI, loyalty intention; RI, recommendation intention; Variables measured on a 7-point Likert scale (1 = lowest; 7 = highest); multiple comparison using *post-hoc* test: ^a^E1 vs. E3, ^b^E1 vs. E4, ^c^E2 vs. E4.

* Significant for *p* < .05.

**Table 6:** Characterization and comparison according to gym attended, in users, of QI, LI and RI.

Dimensions	G1 (*n* = 75)	G2 (*n* = 97)	G3 (*n* = 182)	G4 (*n* = 128)	G5 (*n* = 107)	*p*	*η* ^2^
QI	5.60 ± 1.36^a^^,^^b^	6.02 ± 1.34^c^^,^^d^^,^^e^	6.49 ± .81^a^^,^^c^^,^^f^	6.46 ± .83^b^^,^^d^^,^^g^	5.50 ± 1.39^e^^,^^f^^,^^g^	.001*	.123
LI	5.38 ± 1.65^a^^,^^b^	5.76 ± 1.55^d^	6.30 ± 1.20^a^^,^^f^	6.18 ± 1.25^b^^,^^d^^,^^g^	5.64 ± 1.46^f^^,^^g^	.001*	.057
RI	5.44 ± 1.62^a^^,^^b^	5.86 ± 1.43^d^	6.48 ± 1.04^a^^,^^f^	6.28 ± 1.23^b^^,^^d^^,^^g^	5.53 ± 1.50^f^^,^^g^	.001*	.091

*η*^2^ = eta squared (effect size); G1, Gym 1; G2, Gym 2; G3, Gym 3; G4, Gym 4; G5, Gym 5; QI, quality of intervention; LI, loyalty intention; RI, recommendation intention; Variables measured on a 7-point Likert scale (1 = lowest; 7 = highest); multiple comparison using *post-hoc* test: ^a^G1 vs. G3, ^b^G1 vs. G4, ^c^G2 vs. G3, ^d^G2 vs. G4, ^e^G2 vs. G5, ^f^G3 vs. G5, ^g^G4 vs. G5.

* Significant for *p* < .05.

There are significant differences, considering only the user's opinion and according to sex (Table 3), regarding RI (*t* = 2.109; *p* = .035; *d* = .205, moderate ES). In the three dimensions, mean answer of female users is higher than those male users [QI: F (6.13 ± 1.18), M (6.04 ± 1.18); LI: F (5.98 ± 1.36), M (5.84 ± 1.60); RI: F (6.10 ± 1.33), M (5.81 ± 1.52)].

According to age, there are significant differences in the three dimensions (Table 4): QI (*F* = 3.596; *p* = .003; *η*^2^ = .030, small ES), LI (*F* = 2.451; *p* = .033; *η*^2^ = .021, small ES) and RI (*F* = 2.573; *p* = .026; *η*^2^ = .022, small ES). The highest answer values were provided by older users, specifically those over 60 years old (QI: 6.53 ± .79; LI: 6.51 ± .90; RI: 6.56 ± .72). The lowest values were reported by users aged 21–30 (QI: 5.87 ± 1.23; LI: 5.79 ± 1.41; RI: 5.85 ± 1.57). By interpreting the results of the *post-hoc* test, in the three dimensions, it is possible to verify that the differences come from A6 (over 60 years old) with A2 (21–30 years old) and A3 (31–40 years old).

In education level, there are significant differences in two dimensions (Table 5): QI (*F* = 3.690; *p* = .012; *η*^2^ = .019, small ES) and LI (*F* = 3.775; *p* = .011; *η*^2^ = .019, small ES). The highest answer rates were provided by users with less scholarlily (9th grade) (QI: 6.49 ± .97; LI: 6.25 ± 1.41; RI: 6.30 ± 1.33) and the lowest by users with a BSc degree (QI: 5.96 ± 1.27; LI: 5.85 ± 1.44) and MSc or PhD (RI: 5.85 ± 1.52). Regarding the origin of the differences between the groups: in QI, the differences result from E1 (9th grade) with E3 (BSc degree); in LI, result from E4 (MSC or PhD) with E1 (9th grade) and E2 (12th grade).

Gym attendance was associated with significant differences in all three dimensions (Table 6): QI (*F* = 20.485; *p* = .001; *η*^2^ = .123, medium ES), LI (*F* = 8.787; *p* = .001; *η*^2^ = .057, medium ES) and RI (*F* = 14.554; *p* = .001; *η*^2^ = .091, medium ES). The highest rates were obtained in G3 (QI: 6.49 ± .81; LI: 6.30 ± 1.20; RI: 6.48 ± 1.04) and the lowest in G5 (QI: 5.50 ± 1.39) and G1 (LI: 5.38 ± 1.65; RI: 5.44 ± 1.62). In QI dimension, the differences result: from G5 with G2, G3 and G4; from G4 with G1 and G2; from G3 with G1 and G2. In the LI and RI dimensions: from G5 with G3 and G4; from G4 with G1 and G2; from G3 with G1.

## 4 Discussion

Although several differences reached statistical significance, the associated effect sizes were generally small to moderate. Therefore, the findings should be interpreted with appropriate caution, emphasizing their practical and applied relevance within real-world fitness service contexts rather than overestimating their magnitude. Overall, users reported high evaluations of the fitness service across all three dimensions. Specifically, the quality of the intervention was rated positively, users indicated strong loyalty intentions, and they expressed a high likelihood of recommending the gym to others. In parallel, instructors tended to estimate lower values for these same dimensions when predicting how users would rate the service. This consistent gap between users' self-reported perceptions and instructors' estimations highlights a systematic underestimation of users' satisfaction by fitness professionals, with potential implications for service management and communication within fitness centers. The age profile of the instructors, compared to users, may partially contribute to this perceptual gap, as generational differences in service expectations and relational dynamics can shape how quality and satisfaction are interpreted. Most of the evidence supporting these relationships is based on users' service quality self-reported perception and related behavioral intentions, whereas the present research extends this literature, by incorporating instructors’ estimations of users' evaluations, offering a complementary comparative perspective.

### 4.1 Perceptual differences between users and instructors' estimations of users' evaluations

Significant perceptual gaps were observed between instructors and users in all dimensions, with user consistently assigning higher ratings. This pattern echoes the conclusions of Franco et al. (36), who reported incongruence between instructors' self-perceptions and users' evaluations of service quality.

A possible explanation lies in the instructors' professional profile, often younger and less experienced, which may lead to self-critical assessments or an underestimation of their own performance (7, 37). Conversely, users may exhibit positivity bias, evaluating services more favorably due to satisfaction or social desirability effects. This discrepancy highlights the importance of reflective training practices and feedback-based evaluation systems to align instructors' self-awareness with user expectations, ultimately improving the quality and consistency of service delivery.

Importantly, this perceptual gap should not be interpreted merely as an individual bias, but rather as a potential structural feature of fitness service delivery. Instructors operate under continuous performance pressure and professional self-scrutiny, which may lead to conservative estimations of user satisfaction. From a theoretical approach, our findings highlight the importance of perceptual alignment as a mechanism that connects service quality, professional behavior, and relationship sustainability in fitness settings.

### 4.2 Variations in users' ratings according to sex, age group, education level, and gym attended

Female users demonstrated significantly higher intentions to recommend their gyms compared to male users. This result echoes findings by Barbosa et al. (22), suggesting that women may be more responsive to relational quality and emotional connection with instructors. Men, by contrast, may adopt a more utilitarian perspective, focusing on functional outcomes rather than relational satisfaction. These distinctions highlight the potential value of gender-sensitive communication strategies and promotional approaches to stimulate positive word-of-mouth.

Age related trends revealed that perceived instructor quality and behavioral intentions increased with age, reaching their highest levels among users over 60 years old. Similar patterns have been reported by Campos et al. (25) and Ortega-Martínez et al. (1), who found that older participants tend to value interpersonal relationships, trust, and perceived competence more strongly. Younger users (particularly those aged 21–30 years) appeared more demanding and less loyal, potentially due to greater exposure to digital fitness platforms, higher expectations for personalization, and lower affective attachment to traditional gym settings (11).

Educational level also emerged as a significant factor. Participants with lower educational attainment (for example 9th or 12th grade) reported higher satisfaction and loyalty intentions than those with university or postgraduate degrees. This finding is consistent with Ferreira-Barbosa et al. (19, 20), who noted that more educated consumers often hold higher standards regarding service innovation, instructor expertise, and communication quality. These results suggest that customer segmentation by educational background could enhance the targeting of communication and engagement strategies.

Substantial inter-gym variability further indicates that contextual and managerial factors influence users' evaluations. Gyms G3 and G4 consistently record higher quality of intervention, loyalty intention, and recommendation intention scores, suggesting that localized culture, instructor stability, and quality management practices can foster a more cohesive service experience. These differences may be partly explained by variations in instructors' interpersonal and communicative skills, which shape the relational climate experienced by members. Gyms where instructors communicate more clearly, demonstrate greater empathy, and build stronger interpersonal connections with users are more likely to promote trust, satisfaction, and long-term loyalty, whereas more transactional or less supportive interaction styles may weaken users' emotional engagement with the service. This interpretation is consistent with Gonçalves et al. (18) and Glaveli et al. (21), who emphasize the central role of interpersonal and communicative skills in user trust, satisfaction, and loyalty.

### 4.3 Theoretical contribution and managerial implications

Overall, this study advances service quality theory in fitness contexts by demonstrating that perceived instructor quality, loyalty intention, and recommendation intention are shaped not only by service delivery itself, but also by systematic perceptual and contextual moderators. From a theoretical standpoint, this study extends traditional service quality and loyalty models in fitness contexts by incorporating instructors' estimations of users' perceptions as a distinct analytical layer. Rather than relying exclusively on users' self-reported evaluations, the findings highlight the relevance of second-order perceptions and perceptual gaps as mechanisms that shape professional self-regulation, service delivery, and relationship sustainability in fitness services. By integrating users' direct evaluations with instructors' estimations of user experience, the findings extend existing service quality models in the fitness industry (4, 12, 21, 25) and provide a more nuanced understanding of the mechanisms linking perceived quality to behavioral outcomes. Moreover, the identification of demographic moderators reinforces theoretical approaches to customer segmentation, behavioral loyalty, and advocacy in sport and exercise settings (14).

The findings provide several practical implications for fitness managers and organizations aiming to enhance service quality, user satisfaction, and long-term loyalty (29, 30). First, investing in instructor development and feedback system is crucial. Structured professional development programs should focus on strengthening interpersonal communication, and self- assessment skills, particularly among younger or less experienced instructors (7–10). Incorporating mechanisms such as peer observation, mentoring, and systematic user feedback help align instructors' self-perceptions with user expectations and improve the overall quality and consistency of the service provided (12, 21, 37).

Second, the results suggest that segmentation-based loyalty strategies can be effective in addressing the specific needs of diverse user groups (14). Younger users, who tend to be more critical and less loyal, may benefit from interactive, technology-integrated experiences that enhances engagement; older users appear to value relational stability, personalized attention, and a sense of belonging (1, 25). Designing loyalty programs that reflect these generational preferences can strengthen satisfaction and behavioral commitment across user segments (21).

Third, continuous monitoring and quality management should be embedded in the operational routines of fitness centers. Regular assessments of instructor quality, loyalty and recommendation intentions through structured surveys and performance review allow for the early identification of service gaps (12, 29, 30). The significant inter-gym differences observed in this study highlight the need for local adaptation, ensuring that management strategies respond to each gym's culture, clients, and operational realities (21). Furthermore, personalized communication between instructors and users should be encouraged. Adapting communication style and service approach according to demographic variables such as age, education and gender can reinforce perceived value and relational trust (14). Instructors who personalize their interactions are more likely to foster loyalty and stimulate recommendation intentions (12, 25).

Promoting a culture of co-creation is essential for sustained engagement. Encouraging instructors to act as active partners in shaping user experience through empathy, dialogue, and responsiveness strengthen user instructor relationships and consolidate long-term loyalty (21, 38). By translating these insights into practice, fitness organizations can build stronger emotional connections with users, improve satisfaction and loyalty, and reinforce their competitive advantage in an evolving increasingly customer-driven market.

### 4.4 Limitations and future research

Despite its contributions, this study presents several limitations that should be acknowledged. Its cross-sectional design limits causal inference, as the observed relationship between instructor quality, loyalty and recommendation intentions represent associations rather than cause-effect links. Future research employing longitudinal or experimental designs could explore how the instructor behaviors and user perceptions change over time and how specific interventions influence loyalty development. In applied fitness service contexts, this may involve repeated assessments at two or three time points throughout the year within the same facilities, allowing the examination of temporal dynamics while accounting for the high membership turnover typical of the industry.

The study was conducted within a single fitness chain in Portugal, which constrains the generalizability of findings. Although, data were collected from five gyms with diverse user profiles, organizational and cultural homogeneity may still have influenced perceptions. Additionally, unequal distribution of participants across user profiles and sociodemographic categories represents a limitation. Although the statistical procedures are robust to differences in group size, smaller subsamples in certain groups may have reduced statistical power and should be interpreted with caution. Given the number of group comparisons conducted, an increased risk of Type I error cannot be ruled out. However, the analytical strategy was intentionally focused on theoretically driven comparisons aligned with the study objectives, and effect sizes were systematically reported to support a cautious interpretation of statistically significant findings. Future investigations with more balanced samples would strengthen comparative analyses and enhance the generalizability of findings. Comparative studies across different chains, business models (e.g., low-cost, boutique, and premium clubs), ownership structures, or cultural settings could validate and extend the proposal to other service.

The use of self-reported questionnaires introduces potential bias, particularly social desirability and overestimation of satisfaction. Additionally, as participation was voluntary, a self-selection bias cannot be ruled out, with the possibility that more engaged or more satisfied users were more likely to respond. Complementary methodologies like direct observations, interviews, or maybe third-party evaluations, could be a more objective measured of service quality and instructor performance. Future research should incorporate additional psychological and experiential constructs, such as emotions experienced after service encounters (e.g., post-class emotional responses in group fitness contexts), alongside motivation, affective commitment, or perceived value, for a deeper understanding of the mechanism linking instructor behavior to user loyalty. Integrating these constructs into structural models, could enhance theoretical explanations and practical applications in exercise service management.

Rather than limiting the contribution of the present study, these aspects open promising avenues for future longitudinal, experimental, and cross-cultural research aimed at better understanding how perceptual alignment between instructors and users evolves over time and influences long-term loyalty and service sustainability.

## 5 Conclusions

The present research contributes to the fitness service literature by highlighting a systematic perceptual gap between users' evaluations and instructors' estimations of service quality, loyalty intention, and recommendation intention. The findings indicate that users tend to evaluate the service more positively than instructors anticipate, underscoring the relevance of perceptual alignment as a key element in fitness service management. By identifying sociodemographic and inter-gym differences in these evaluations, the study provides actionable insights for managers to tailor communication strategies, enhance instructor training, and develop more effective loyalty-oriented service models.

The findings further demonstrate that sociodemographic factors play a relevant role in shaping users' perceptions, particularly age and educational level, with older and less-educated users consistently reporting higher evaluations of the service quality, loyalty, and recommendation intentions. The sex differences were limited to recommendation intention, favoring female users, while significant inter-gym variations highlight the importance of contextual and organizational factors.

From a theoretical perspective, this study advances the understanding of service quality in fitness contexts by showing how perceptions of instructor quality translate into loyalty and recommendation intentions and by identifying demographic and contextual moderators of these relationships. By integrating both user and instructor perspectives, the findings reveal a systematic perceptual gap that underscores the importance of reflective professional development and user-centered service management. From a practical standpoint, the results inform strategies for personalized, relationship-oriented service delivery, reinforcing the central role of instructor quality in sustaining user satisfaction, loyalty, and long-term organizational success.

## Data Availability

The original contributions presented in the study are included in the article/Supplementary Material, further inquiries can be directed to the corresponding authors.
