# Correction: Perceptions of instructor quality, loyalty and recommendation intentions in fitness centers: a comparative analysis by role and users characteristics

**DOI:** 10.3389/fspor.2026.1901230

**Published:** 2026-06-30

**Authors:** Francisco Campos, Daniela Almeida, José Aleixo, Diogo Aveiro, Viorel Petru Ardelean, Vlad Adrian Geantă, Fernando Martins

**Affiliations:** 1 Polytechnic University of Coimbra, Coimbra, Portugal; 2 SPRINT – Sport Physical activity and health Research & INnovation cenTer, Polytechnic University of Coimbra, Coimbra, Portugal; 3 Instituto de Telecomunicações – Delegação da Covilhã, Covilhã, Portugal; 4 LFitness, Oliveira do Bairro, Portugal; 5 Department of Physical Education and Sport Sciences, University of Limerick, Limerick, Ireland; 6 Department of Physical Education and Sport, Faculty of Physical Education and Sport, Aurel Vlaicu University of Arad, Arad, Romania; 7 inED – Center for Research and Innovation in Education, Polytechnic University of Coimbra, Coimbra, Portugal

**Keywords:** fitness centers, fitness service, loyalty, perceived quality, recommendation intention

Affiliation 1, Polytechnic University of Coimbra, Coimbra, Portugal, was erroneously given as Department of Education, Sports and Social Intervention, Polytechnic University of Coimbra, Coimbra, Portugal.

Author Fernando Martins was erroneously assigned to affiliation 8, Department of Training of Educators and Teachers, Polytechnic University of Coimbra, Coimbra, Portugal. This affiliation has now been removed.

The original version of this article has been updated.

